# Rosuvastatin exerts anti-atherosclerotic effects by improving macrophage-related foam cell formation and polarization conversion via mediating autophagic activities

**DOI:** 10.1186/s12967-021-02727-3

**Published:** 2021-02-10

**Authors:** Xinxin Zhang, Yating Qin, Xiaoning Wan, Hao Liu, Chao Lv, Weibin Ruan, Lin He, Li Lu, Xiaomei Guo

**Affiliations:** 1 Department of Cardiology, Tongji Hospital, Tongji Medical College, Huazhong University of Science and Technology, Wuhan, 430030 China; 2 Bio-X Institutes, Key Laboratory for the Genetics of Developmental and Neuropsychiatric Disorders (Ministry of Education), Shanghai Jiaotong University, Shanghai, China; 3 Department of Cardiology, Renmin Hospital of Wuhan University, Wuhan, Hubei China

**Keywords:** Atherosclerosis, Rosuvastatin, Autophagy, Lipid accumulation, Macrophage polarization

## Abstract

**Background:**

Atherosclerosis is a chronic vascular disease posing a great threat to public health. We investigated whether rosuvastatin (RVS) enhanced autophagic activities to inhibit lipid accumulation and polarization conversion of macrophages and then attenuate atherosclerotic lesions.

**Methods:**

All male Apolipoprotein E-deficient (ApoE^−/−^) mice were fed high-fat diet supplemented with RVS (10 mg/kg/day) or the same volume of normal saline gavage for 20 weeks. The burden of plaques in mice were determined by histopathological staining. Biochemical kits were used to examine the levels of lipid profiles and inflammatory cytokines. The potential mechanisms by which RVS mediated atherosclerosis were explored by western blot, real-time PCR assay, and immunofluorescence staining in mice and RAW264.7 macrophages.

**Results:**

Our data showed that RVS treatment reduced plaque areas in the aorta inner surface and the aortic sinus of ApoE^−/−^ mice with high-fat diet. RVS markedly improved lipid profiles and reduced contents of inflammatory cytokines in the circulation. Then, results of Western blot showed that RVS increased the ratio LC3II/I and level of Beclin 1 and decreased the expression of p62 in aortic tissues, which might be attributed to suppression of PI3K/Akt/mTOR pathway, hinting that autophagy cascades were activated by RVS. Moreover, RVS raised the contents of ABCA1, ABCG1, Arg-1, CD206 and reduced iNOS expression of arterial wall, indicating that RVS promoted cholesterol efflux and M2 macrophage polarization. Similarly, we observed that RVS decreased lipids contents and inflammatory factors expressions in RAW264.7 cells stimulated by ox-LDL, accompanied by levels elevation of ABCA1, ABCG1, Arg-1, CD206 and content reduction of iNOS. These anti-atherosclerotic effects of RVS were abolished by 3-methyladenine intervention. Moreover, RVS could reverse the impaired autophagy flux in macrophages insulted by chloroquine. We further found that PI3K inhibitor LY294002 enhanced and agonist 740 Y-P weakened the autophagy-promoting roles of RVS, respectively.

**Conclusions:**

Our study indicated that RVS exhibits atheroprotective effects involving regulation lipid accumulation and polarization conversion by improving autophagy initiation and development via suppressing PI3K/Akt/mTOR axis and enhancing autophagic flux in macrophages.

## Background

Atherosclerosis is characterized by ascensive buildup of plaque in the arterial wall with perturbation of lipid metabolism and vascular inflammation [1]. Endothelium dysfunction results in subendothelial accumulation of oxidized low-density lipoprotein (ox-LDL), and transmigration of monocytes into arterial wall where they differentiate into macrophages that engulf excessive ox-LDL to generate lipid-laden foam cells, which ultimately triggers the initiation and development of atherosclerosis [1–3]. Besides, macrophages produce pro-inflammatory mediators under ox-LDL stimulation, which aggravates the atherosclerotic lesions [4, 5].

Macrophages, an immune cell population with high heterogeneity in phenotype and function, can alter own polarization state to adapt to complex external conditions [6–9]. When exposed to pro-inflammatory substances, resting macrophages turn to develop classical M1 phenotype capable of producing inflammatory factors including IL-6, IL-1β, TNF-α, and iNOS, leading to inflammation amplification and atherosclerosis development [10–12]. On the contrary, the alternatively activated M2 macrophages are generated in response to IL-4 or IL-13, which release anti-inflammatory factors such as IL-10, Arginase-1 and then possess anti-atherosclerotic ability [13, 14].

Autophagy, a pro-survival intracellular process, has been demonstrated to attenuate burgeoning plaques via suppressing foam cell formation and weakening inflammatory response [15, 16]. It is reported that pro-atherosclerotic factors such as ox-LDL could block autophagic cascades, whereas up-regulated autophagy pathway effectively blunts atherosclerosis progression in vitro and in vivo [17–20]. It has been suggested that autophagy displays important roles in M2 macrophage formation and there exists an association of macrophage polarization with autophagy signal transduction. Phosphoinositide3-kniase (PI3K)/Akt/mammalian target of rapamycin (mTOR) pathway serves as a major regulator inhibiting the initiation of autophagic activities [21, 22]. Moreover, inhibition of PI3K/Akt/mTOR pathway has been shown to improve cholesterol efflux capacity of macrophage-derived foam cells and weaken atherosclerotic plaque inflammation by enhancing autophagy [21, 23]. mTOR inhibitor rapamycin has been indicated to play beneficial roles in alleviating development of plaque lesions [24].

Statins are inhibitors of 3-hydroxyl-3-methylglutaryl coenzyme A (HMG-CoA) reductase, responsible for synthesis of endogenous cholesterols [25]. Statins alleviate atherosclerosis associated with lowering circulating cholesterol, modulation of angiogenesis and its anti-inflammatory, antioxidant properties [26, 27]. Rosuvastatin (RVS), a kind of HMG-CoA reductase inhibitor, has been reported to ameliorate atheroma lesions via improving foam cell formation and inflammation reaction [28, 29]. Clinically, RVS possesses potent lipid-lowering actions and has been broadly used for the prophylaxis and treatment of atherosclerosis and subsequent complications including coronary heart disease and ischemic stroke. For instance, a recent study showed that RVS is effective in stabilizing non-obstructive neoatherosclerotic plaques [30]. Besides, RVS has been demonstrated to significantly decrease LDL-C level in children with homozygous familial hypercholesterolemia [31]. Thondapu and colleagues reported that treatment of RVS improved lipid profiles and induced obviously plaque stabilization and regression of plaque volume in patients with coronary artery disease [32]. However, the upstream molecular mechanisms by which RVS exerts these atheroprotective actions remains to be explored. In this study, we investigated the effects of RVS on atherosclerosis development in vivo and in vitro and discovered that RVS intervention significantly attenuated the atherosclerotic lesions, which was attributed to RVS-induced regulation of macrophages lipid accumulation and polarization conversion by enhancing autophagy via mediating PI3K/Akt/mTOR pathway and autophagic flux.

## Material and methods

### Drugs

RVS used in this study was granted from AstraZeneca (Shanghai, China).

### Animal study

This study process was performed according to the National Institutes of Health Guide for the Care and Use of Laboratory Animals and was approved by the Institutional Animal Care and Use Committee of Tongji Medical College, Huazhong University of Science and Technology, Wuhan, China.

All 8-week-old ApoE^−/−^ mice were purchased from Beijing Vital River Experimental Animal Technology Co, Ltd. (Beijing, China) and were acclimatized to environment with ad libitum access to food and water for 2 weeks, then randomly subdivided into two groups. The mice were fed with high-fat diet of 21% fat supplemented with 0.15% cholesterol. The mice in RVS group were orally gavaged with RVS at a dose of 10 mg/kg/day, and the control group were treated with the same volume of normal saline for 20 weeks (n = 12 each group). At the end of the experiment, all mice were fasted overnight and were anaesthetized. Then the blood samples were harvested via cardiac puncture, and heart and aorta tissues were obtained for histologic and molecular mechanism detections.

### En face Oil Red O (ORO) staining

En face plaque area was commonly examined by ORO (ORO, St. Louis, USA) staining of lipid deposits. The entire aorta was dissected from mice free of fat and connective tissues, then unfolded, opened longitudinally and soaked in 4% paraformaldehyde overnight. Subsequently, the whole aorta was stained with 0.5% ORO solution and photographed with a digital camera (Olympus Corporation, Japan).

### Histological, morphometric analysis and immunofluorescence staining

After fixed in 4% paraformaldehyde for 24 h, the mouse heart and aorta tissues were soaked in optimum cutting temperature compound and were kept at − 80 °C. Then serial cryosections (8 μm) were harvested and ORO staining of these sections was performed to detect the lipid deposition within the aortic sinus plaque.

After excision and fixation in 4% paraformaldehyde solution, the heart and ascending aorta was washed, dehydrated and embedded in paraffin. The aortic sinus sections at 5 μm thickness were obtained with a slicing machine for next step. Histological analysis of aortic sinus was carried out using hematoxylin and eosin (HE, Beyotime, Beijing, China) staining following published protocols to evaluate the size of atherosclerotic plaques. Moreover, immunofluorescence measurement was performed to assess the level of LC3II within aortic lesions. In short, the sections were incubated with anti-LC3II antibody (1:200, Abcam, Rabbit IgG) at 4 °C overnight, then incubated with an FITC conjugated goat anti-rabbit secondary antibody (1:100, Servicebio Biotechnology, Wuhan, China) and mounted with DAPI (1:200, Servicebio Biotechnology, Wuhan, China). Then the level of LC3II was analyzed with a fluorescence microscope (Olympus Corporation, Japan).

### Measurement of blood lipid profiles

After centrifugation of 3000 rpm for 10 min, supernatants of blood samples were stored at − 80 °C. The circulating concentrations of triglyceride (TG), total cholesterol (TC), low density lipoprotein cholesterol (LDL-C) and high density lipoprotein cholesterol (HDL-C) were measured using corresponding commercial kits according to the manufacturer's instructions (Jiancheng Bioengineering Institute, Nanjing, China).

### Detection of serum cytokines

Serum cytokines including CRP, TNF-α, IL-1β and IL-6 were determined using enzyme-linked immunosorbent assay (ELISA, Boster Biological Technology, China) following manufacturer’s protocols.

### Cell culture and treatment

RAW264.7 murine macrophages were obtained from American Type Culture Collection (ATCC, Manassas, VA, USA) and cultured in Dulbecco's modified Eagle medium (DMEM) supplemented with 10% fetal bovine serum (Gibco, Grand island, NY, USA) in a humidified incubator with 5% CO2 at 37℃. RAW264.7 cells were seeded in 6-well plates and then treated with ox-LDL at a concentration of 50 ug/ml (Yiyuan Biotechnologies, China) with or without RVS (5, 10, 25 µM) for 24 h. Cells were co-cultured with 3-methyladenine (3-MA; 5 mM, Selleckchem, Houston, USA) or rapamycin (Rap, 10 nM, MedChemExpress, USA) to impede or activate autophagy aimed at ensuring the role of autophagy. Cells were pretreated with chloroquine (CQ; 20 μM, Selleckchem, Houston, USA) to examine the effects of RVS on disturbed autophagy flux. LY294002 (10 μM, Selleckchem, Houston, USA) and 740 Y-P (10 μM, MedChemExpress, USA) was separately added to cells to investigate the roles of PI3K/Akt/mTOR pathway. After different interventions, RAW264.7 cells were harvested and subjected to subsequent experiments.

### Oil Red O (ORO) staining

After different interventions, cultured RAW264.7 cells were washed three times with PBS, fixed with 4% paraformaldehyde for 10 min and then washed twice again, followed by staining with ORO working solution for 30 min. Then, the cells were washed three times with PBS. Lipid droplets in cells were stained in red color and scanned using a scanning microscope. Besides, the absorbance of intracellular lipid droplets was detected at 520 nm.

### Immunofluorescence staining

RAW264.7 macrophages were washed with PBS, fixed with 4% paraformaldehyde and subsequently permeabilized using 0.3% Triton X-100. After blocking with 5% BSA, the cells were incubated with anti-LC3II (1:100, Proteintech Biotechnology, Chicago, USA), or anti-Arg-1 (1:100, Mouse IgG, Cell Signaling Technology, Boston, USA) and anti-iNOS (1:200, Rabbit IgG, Cell Signaling Technology, Boston, USA) antibodies overnight at 4 °C. Subsequently, cells were rinsed with PBS, then incubated with an FITC conjugated goat anti-rabbit secondary antibody (1:200, Servicebio Biotechnology, Wuhan, China) or Cy3 conjugated goat anti-mouse secondary antibody (1:200, Servicebio Biotechnology, Wuhan, China) and mounted with DAPI (1:200, Boster Biological Technology, China). Finally, the cells were photographed using a fluorescence microscope (Olympus Corporation, Japan).

### qRT-PCR

Total RNA of aorta tissues and macrophages was isolated using TRIzol reagent. The cDNA synthesis from total RNA (1 μg) was performed using the ABScript II reagent kit (Abclonal, Boston, USA) following to the manufacturer's protocol. Next, qRT-PCR was carried out on a Roche LightCycler480 System (Roche, USA) using SYBR Green RT-qPCR reagent kit (Abclonal, Boston, USA) with the following primers in Table 1 and results were normalized to the expression level of GAPDH mRNA.

**Table 1:** Primers used for qRT-PCR analysis in this study

Gene	Forward primer sequence (5′-3′)	Reverse primer sequence (5′-3′)
IL-10	CAGAGCCACATGCTCCTAGA	TGTCCAGCTGGTCCTTTGTT
Arg-1	TGCATATCTGCCAAAGACATCG	TCCATCACCTTGCCAATCCC
CD206	TGCTACTGAACCTCCTCAACTGC	AGCCTGACCCCAACTTCTCGT
TNF-α	TCTTCTCATTCCTGCTTGTGG	GGTCTGGGCCATAGAACTGA
IL-6	GGAGCCCACCAAGAACGATAG	GTGAAGTAGGGAAGGCCGTG
iNOS	CCCTTCAATGGTTGGTACATGG	ACATTGATCTCCGTGACAGCC
GAPDH	TGTGAACGGATTTGGCCGTA	GATGGGCTTCCCGTTGATGA

### Western blot

The protein levels of aorta tissues and RAW264.7 macrophages were examined using western blot. The protein was extracted by lysing the aortic tissues and cells with lysis buffer supplemented with 1% protease inhibitors and phosphatase inhibitors (MedChemExpress, USA) on ice. After centrifugation, the supernatant was collected and quantified the concentration using a BCA kit (Boster Biological Technology, Wuhan, China). Then the equal amount of proteins (20 μg) were separated by 10%-12% SDS-PAGE and transferred to PVDF membranes (Millipore). After blocking with 5% non-fat milk, the membranes were probed with different primary antibodies overnight at 4℃, including PI3K, p-PI3K (Tyr 458), Akt, p-Akt (Ser 473), mTOR, p-mTOR (Ser 2448), ULK1, p-ULK1 (Ser 317), p70S6 kinase (S6K), p-S6K (Thr389), S6, p-S6 (Ser235/236), p62, iNOS (Cell Signaling Technology, Boston, USA), LC3 (Proteintech Biotechnology, Chicago, USA), Beclin1, ABCA1, ABCG1 (Abcam, Cambridge, UK), Arg-1, CD206, and GAPDH, β-actin (Abclonal, Boston, USA). Subsequently, the membranes were incubated with horseradish peroxidase-conjugated secondary antibodies (Boster Biological Technology, Wuhan, China). The proteins bands were visualized using ECL kit (Boster Biological Technology, Wuhan, China) and analyzed with Image J software (NIH, USA).

### Statistical analysis

Data in this study were expressed as the mean ± standard deviation (SD) and were analyzed using SPSS 21.0 software version (IBM, Chicago, USA). The significance for differences between two groups were examined using Student's unpaired t test. The differences between multiple groups were measured by One-way ANOVA analysis. The level of p < 0.05 was considered as statistically significance.

## Results

### RVS displayed regulatory effects on plaque burden in ApoE^−/−^ mice

**Fig. 1:**
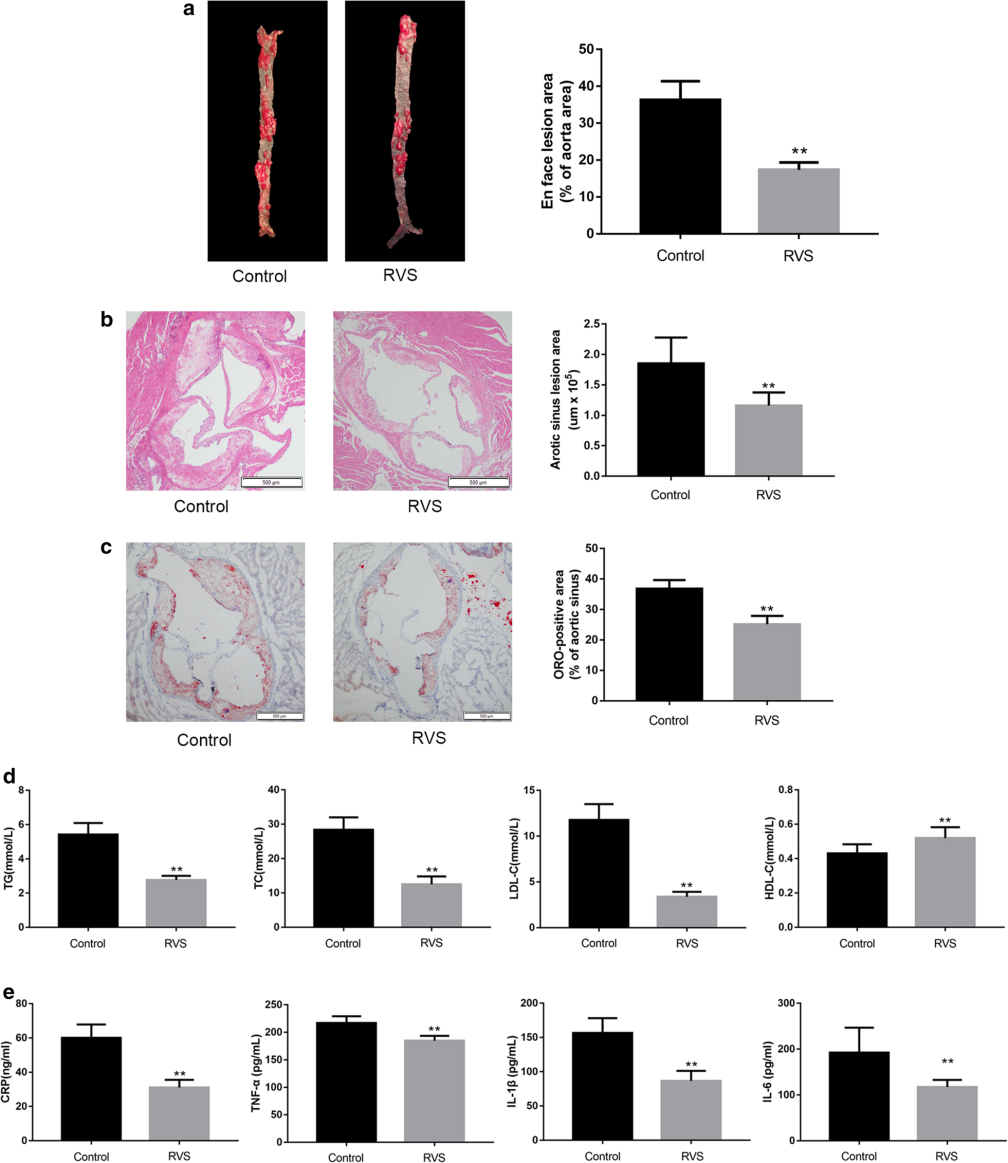
The effects of RVS on the progress of atherosclerotic plaques, blood lipid parameters and the content of inflammatory cytokines in vivo. **a** The lesion area of entire aorta was measured via ORO staining. **b** The size of atheroma plaques in cross-sections of aortic sinus was detected by HE staining. **c** The representative lipid deposition in cryosections of aortic sinus were stained with ORO. **d** The serum level of TG, TC, LDL-C and HDL-C was examined by the corresponding kits. **e** The level of CRP, TNF-α, IL-1β and IL-6 was assayed by ELISA kits. Data were expressed as the mean ± SD, n = 6. *p < 0.05, **p < 0.01 vs. the Control group

At first, we examined the size of plaque area in the whole aorta of the ApoE^−/−^ mice to evaluate the effects of RVS on atherosclerosis development. As shown in Fig. 1a, the extent of atherosclerotic lesions was significantly reduced in RVS treated group compared with the control group (p < 0.01). Moreover, HE-stained transverse sections of mice treated with RVS displayed smaller necrotic core in the aortic sinus (Fig. 1b, p < 0.01), and the results of ORO staining showed RVS decreased lipid deposition in the atherosclerotic plaques (Fig. 1c, p < 0.01).

### RVS effectively alleviated dyslipidemia and inflammation

As lipid dysbolism and inflammation response were involved in the progression of plaque lesions, we measured the levels of lipid profiles and pro-inflammatory cytokines in the circulation of ApoE^−/−^ mice. As described in Fig. 1d, mice with RVS administration had lowered levels of circulating TC (p < 0.01), TG (p < 0.01) and LDL-C (p < 0.01), whereas elevated HDL-C (p < 0.01) level. After 20 weeks of drug intervention, the levels of circulating CRP (p < 0.01), TNF-α (p < 0.01), IL-1β (p < 0.01) and IL-6 (p < 0.01) in RVS group were dramatically reduced compared to the control group (Fig. 1e).

### RVS administration promoted cholesterol efflux and macrophage polarization to an M2 phenotype in the vascular wall

**Fig. 2:**
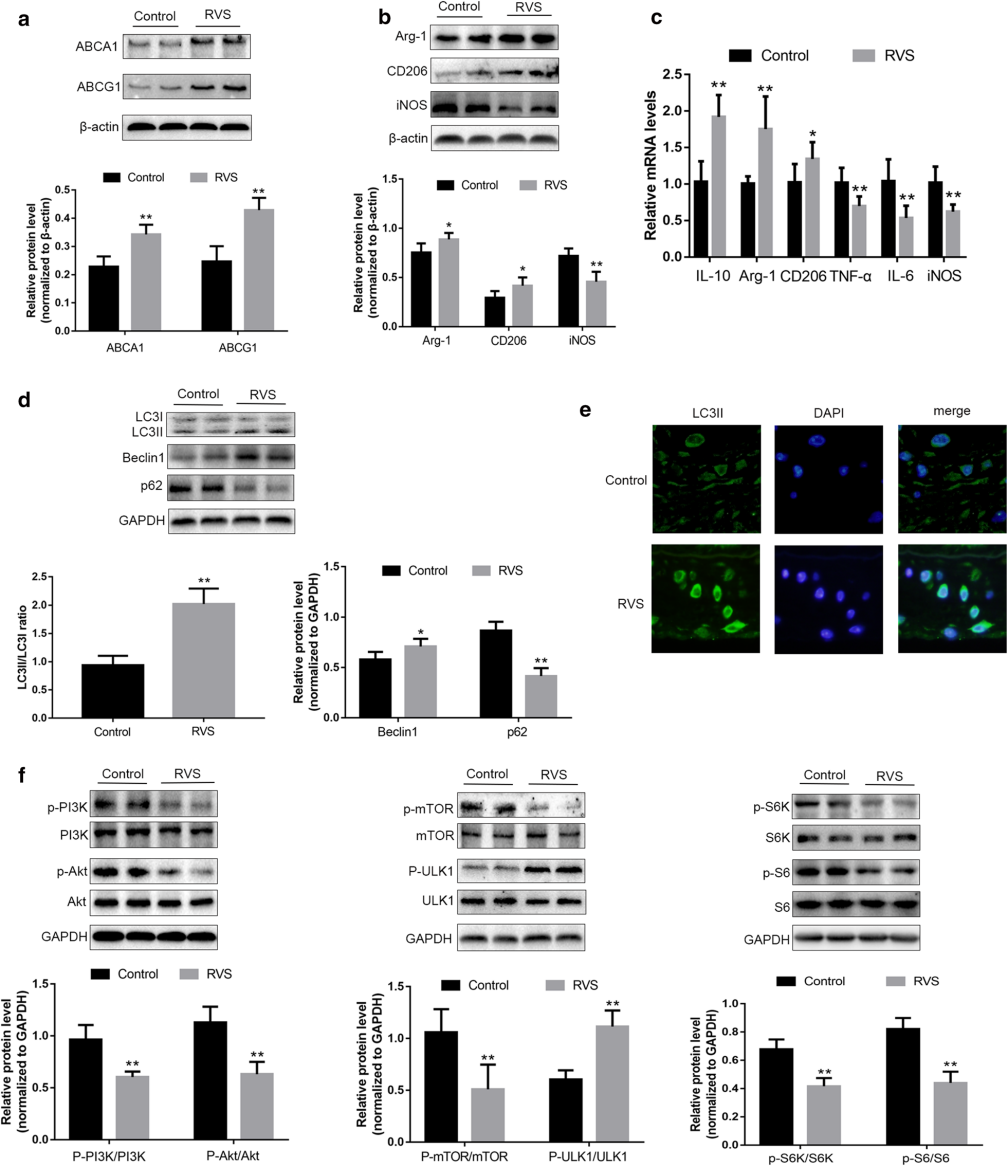
The roles of RVS on the markers of cholesterol efflux and macrophage polarization, autophagy activity and the PI3K/Akt/mTOR pathway in the aortic tissues. **a** The molecules related to lipid efflux from the aorta were detected by western blot. **b** The proteins associated with macrophage polarization were measured by western blot. **c** qRT-PCR was used to examine the mRNA levels of macrophage polarization markers. **d** The molecules associated with autophagy were assayed by western blot. **e** Representative images of immunofluorescence staining of LC3II expression of aortic tissues, scale bar = 50 μm.** f** The molecules linked to PI3K/Akt/mTOR pathway were examined by western blot. Data were expressed as the mean ± SD, n = 6. *p < 0.05, **p < 0.01 vs. the Control group

ABCA1 and ABCG1 were considered to be responsible for reverse cholesterol transport, a process which ameliorated lipid deposition and then perturbed atherosclerotic progression. We next evaluated the effects of RVS on ABCA1 and ABCG1 expression in aorta of ApoE^−/−^ mice. As shown in Fig. 2a, RVS treatment markedly increased the expression of ABCA1 and ABCG1, which confirmed that RVS-induced reduction of lipid droplets accumulation in plaque lesions might be attributed to the increased activities of cholesterol efflux.

On the other hand, we detected the levels of macrophage polarization markers in the aortic tissues. Our results indicated that the expression of iNOS was weakened in the RVS group, whereas the levels of Arg-1 and CD206 were significantly increased after RVS treatment (Fig. 2b). Moreover, we also examined the mRNA levels of M1 and M2 macrophage markers and cytokines produced by different macrophage phenotype. As shown in Fig. 2c, the mRNA levels of M1-related markers iNOS, TNF-α and IL-6 in the vascular wall of the RVS group were obviously diminished, while the expressions of M2-related markers Arg-1, CD206, IL-10 were elevated after RVS intervention. These results indicated that RVS treatment was possibly capable of polarizing macrophages to an M2-like phenotype in the arterial wall.

### RVS enhanced the autophagy process by inhibiting activation of PI3K/Akt/mTOR signaling pathway in ApoE^−/−^ mice

To further explore the detailed mechanisms by which RVS affected cholesterol efflux and macrophage polarization, we evaluated the autophagy activities by detecting the levels of Beclin1, p62 and LC3II/I ratio in the aortic tissues of the ApoE^−/−^ mice. LC3II played an important role in autophagosome formation, and an increased p62 level was closely related to autophagy impairment. As shown in Fig. 2d, RVS significantly increased the content of Beclin1 and the ratio of LC3II/I, whereas reduced p62 level. Additionally, the immunofluorescence staining revealed that RVS treatment obviously enhanced the LC3II expression in the plaque areas, which indicated that RVS treatment ameliorated impaired autophagy induced by high-fat diet (Fig. 2e).

The PI3K/Akt/mTOR pathway possessed an important role in regulating autophagy initiation. The high-fat diet-induced increase in the expression of PI3K, Akt and mTOR in the vascular wall were markedly reduced by RVS administration (Fig. 2f). In addition, RVS treatment significantly up-regulated the phosphorylated level of ULK1 (unc-51-like kinase 1). ULK1 is a serine/threonine protein kinase acting as the crucial member of ULK1 complex. There is evidence showing that the ULK1 complex consists of ULK1 itself, ATG 13 (autophagy-related protein 13), ATG 101 and FIP200 (focal adhesion kinase family interacting protein of 200KD) [33]. It is indicated that PI3K/Akt/mTOR cascade serves as the vital upstream factor which negatively mediates the activation of ULK1 [33]. Once the signaling transduction of PI3K/Akt/mTOR axis is inhibited, the activity of ULK1 is enhanced and then the ULK1 complex participate in the processes of autophagy initiation [33]. Besides, RVS intervention decreased the phosphorylated level of S6K and S6 compared with the control group (Fig. 2f). S6K and eukaryotic inhibition factor 4E-binding protein 1 (4E-BP1) are downstream molecules and activated by mTOR [34]. Phosphorylated S6K is followed by activation of S6, a downstream ribosomal protein and an alternative marker for mTOR activity. S6K and S6 are both attributed to translation initiation and control protein synthesis [34]. These in vivo observations suggested that RVS treatment might facilitate autophagic processes by inhibiting the PI3K/Akt/mTOR pathway, thus contributing to the implementation of anti-atherosclerotic actions of RVS.

### RVS displayed beneficial effects on weakening lipid accumulation and favoring M2-macrophage polarization of RAW264.7 cells stimulated by ox-LDL

**Fig. 3:**
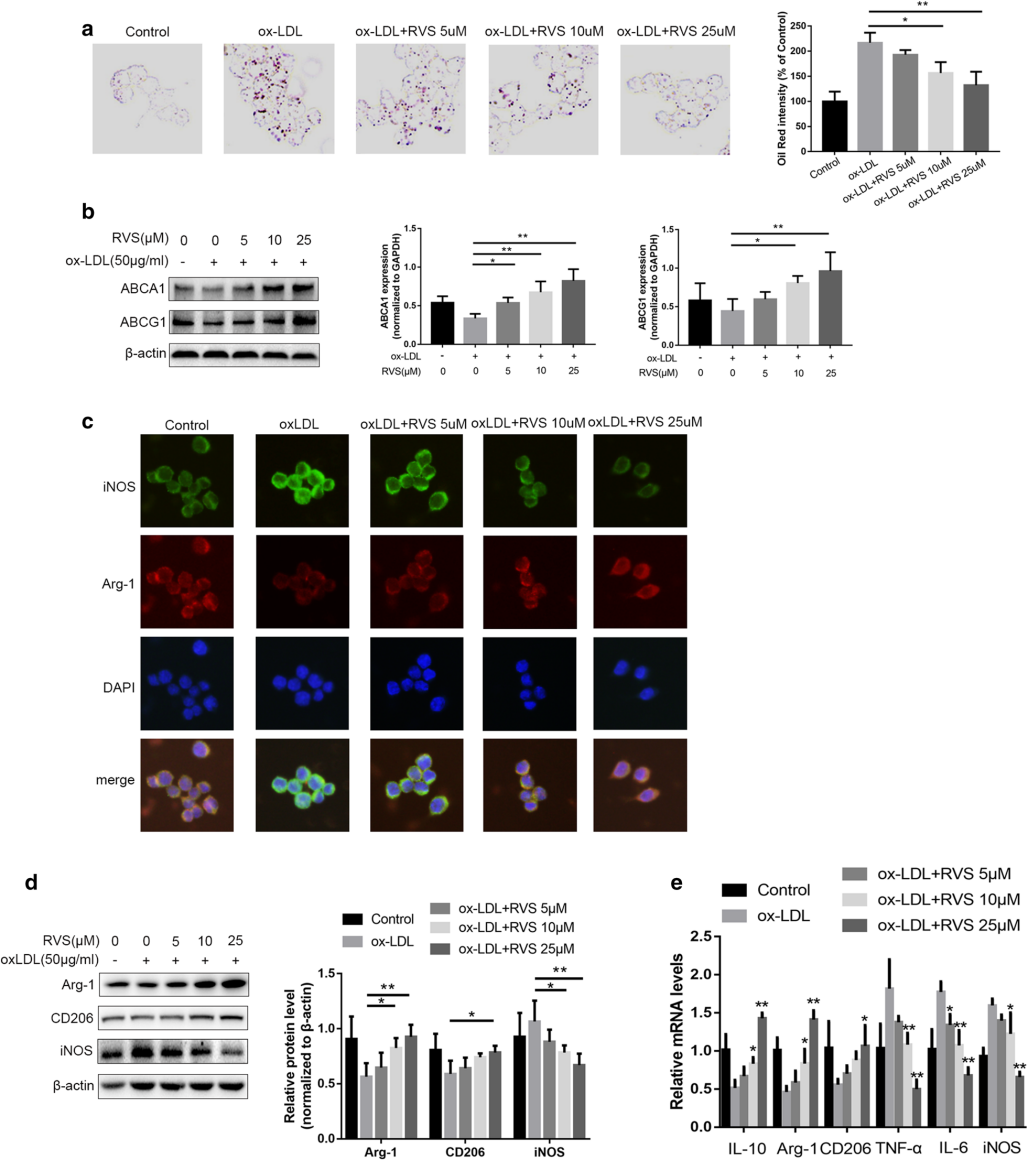
The regulated effects of RVS on lipid deposition and macrophage polarization of RAW264.7 cells stimulated by ox-LDL. **a** The ORO staining was performed to test the lipid deposition of RAW264.7 cells. **b** The expression of bioactive factors responsible for lipid efflux from RAW264.7 cells were examined by western blot. **c**, **d** The level of iNOS, Arg-1 and CD206 in RAW264.7 cells was displayed by immunofluorescence staining and western blot, scale bar = 50 μm. **e** The mRNA level of macrophage polarization markers in RAW264.7 cells. Data were expressed as the mean ± SD from three independent experiments. Four randomly selected visual fields were counted in each group. *p < 0.05, **p < 0.01

Subsequently, to evaluate the effects of RVS on lipid deposition in vitro, we used ox-LDL-induced foam cell formation as the model. As shown in Fig. 3a, the results and ORO Red staining and ORO Red intensity revealed that RVS markedly reduced lipid droplets deposition in RAW264.7 cells and then attenuated foam cell formation. Then we discovered that RVS possibly improved foam cell formation via enhancing the efflux process of lipids within macrophages, as evidenced by RVS-induced increase of ABCA1 and ABCG1 protein abundance in RAW264.7 in a concentration-dependent way (Fig. 3b).

In addition, immunofluorescence staining, western blot and RT-qPCR were performed for determining the expression levels of biomarkers and generated cytokines of RAW264.7 cells treated with or without RVS in the presence of ox-LDL. RVS administration polarized the macrophages from M1-like phenotype induced by ox-LDL to M2-like phenotype, as evidenced by the decreased level of iNOS and increased content of Arg-1 and CD206 in cells with RVS intervention (Fig. 3c, d). What's more, our results showed that RVS remarkably weakened the expression of M1-synthesized TNF-α and IL-6 and elevated the expression of M2-generated IL-10 level (Fig. 3e).

### RVS mediated the autophagy-related processes of RAW264.7 cells insulted by ox-LDL

**Fig. 4:**
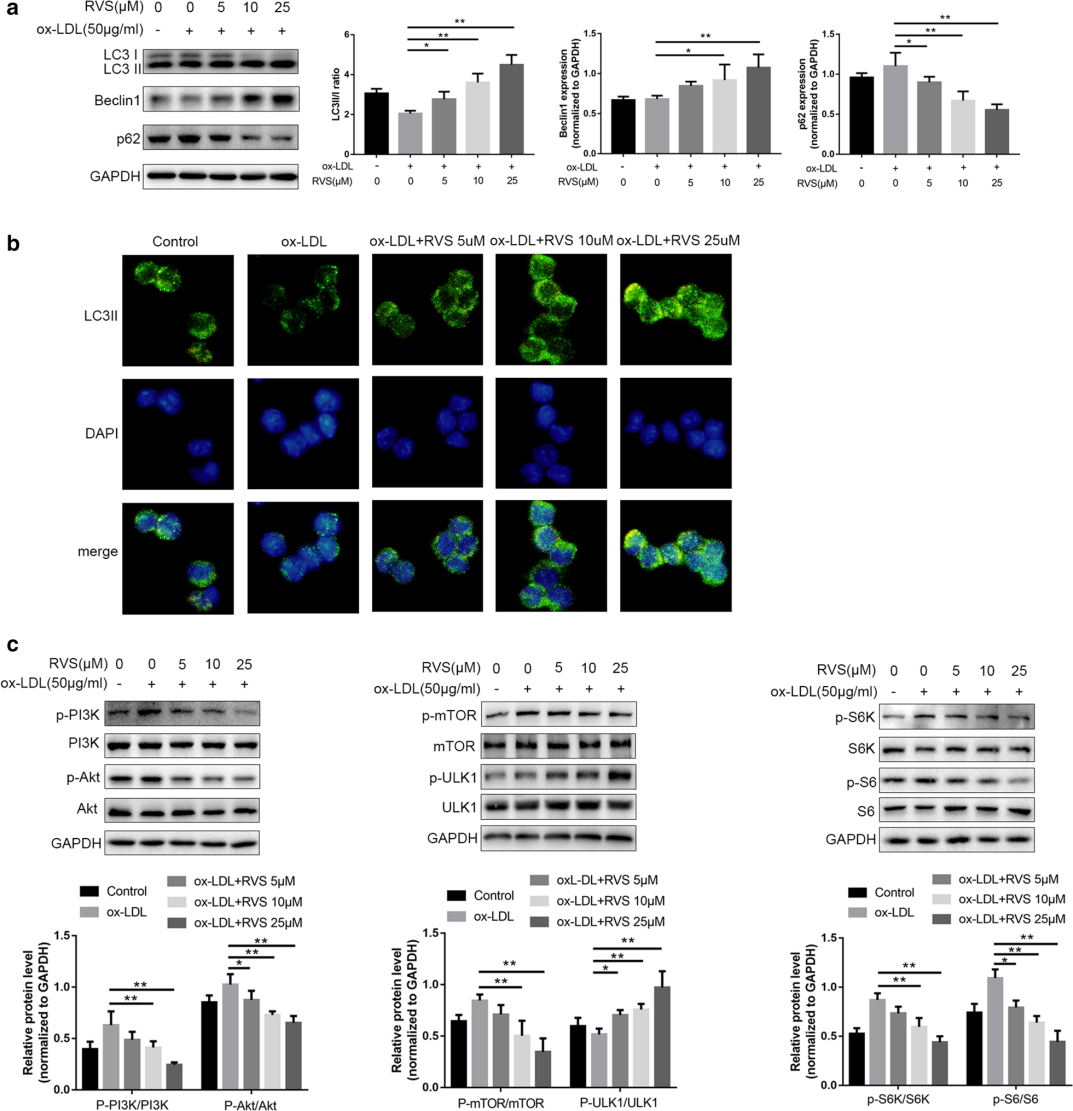
RVS mediated the autophagy-related processes of RAW264.7 cells exposed to ox-LDL. **a** Autophagy-related molecules expressions were measured by western blot. **b** Immunofluorescence staining was performed to detect the LC3II level of RAW264.7 cells, scale bar = 50 μm. **c** The activation of PI3K/Akt/mTOR cascade and ULK1 in RAW264.7 cells. Data were expressed as the mean ± SD from three independent experiments. Four randomly selected visual fields were counted in each group. *p < 0.05, **p < 0.01

Several studies reported that ox-LDL was a blocker of autophagy flux [35–37]. As shown in Fig. 6a, oxLDL stimulation triggered the decrease of LC3II/I ratio and the increase of p62 expression in RAW264.7 cells, which suggested that autophagy process was impeded in the macrophages. Results of western blot and immunofluorescence staining showed that RVS intervention obviously elevated Beclin1 expression, and the ratio of LC3II/I and decreased p62 protein abundance in a dose-dependent manner in RAW264.7 cells stimulated by ox-LDL, indicating that RVS rescued the impaired autophagy activities (Fig. 4a, b). Moreover, we discovered that ox-LDL facilitated the signal transduction of PI3K/Akt/mTOR pathway, followed by the activation inhibition of ULK1 in RAW264.7 cells (Fig. 4c). Whereas, RVS administration effectively suppressed the activation of PI3K/Akt/mTOR cascade and then restored the activity of ULK1 in a dose-dependent way. In addition, RVS treatment decreased the phosphorylated level of S6K and S6 in a dose-dependent manner.

### RVS improved lipid accumulation and polarization conversion of macrophages by regulating autophagy

**Fig. 5:**
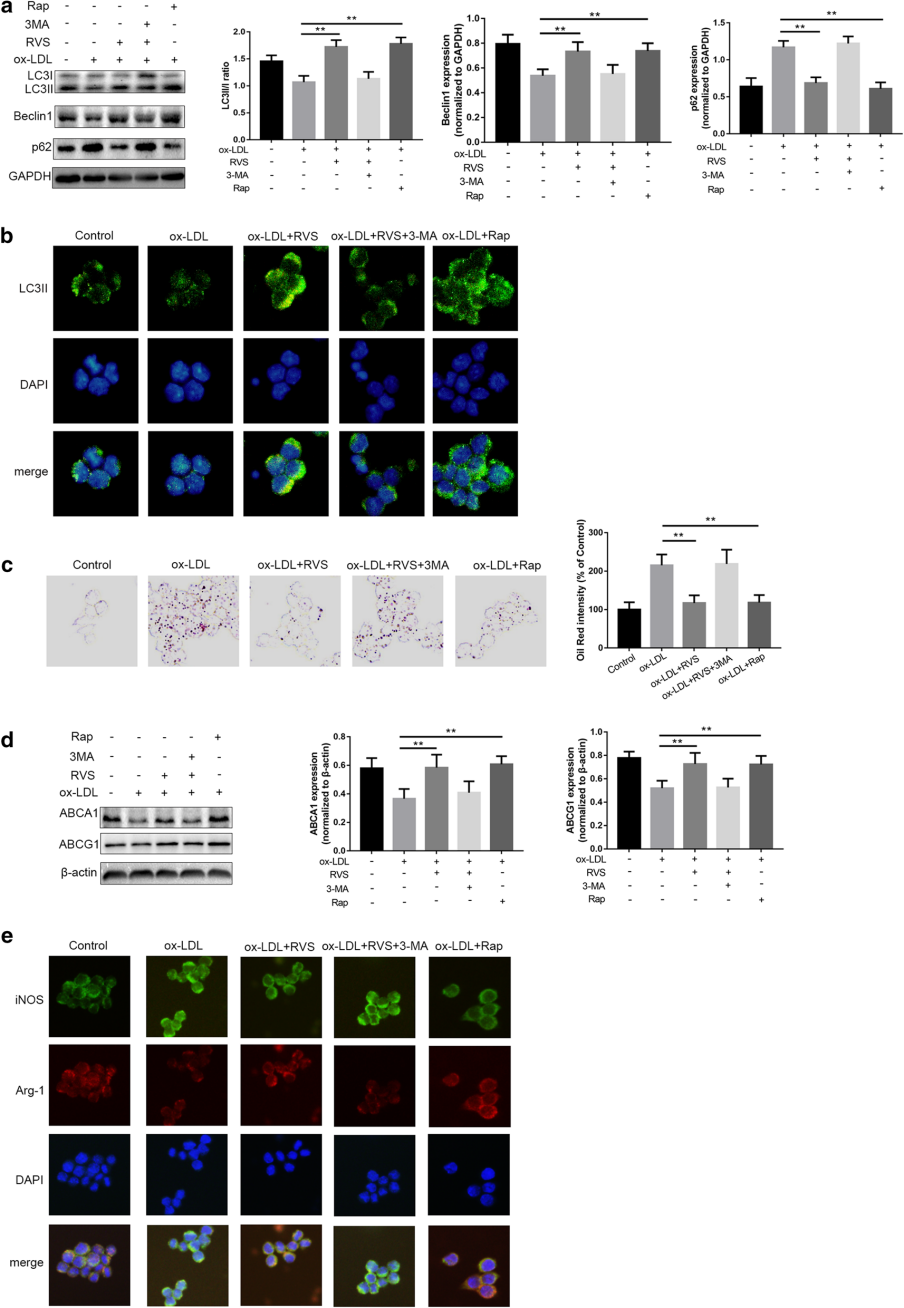
RVS regulated lipid deposition and polarization shift of macrophages via mediating autophagy. **a** Western blot analysis of the levels of autophagy-related molecules. **b** Immunofluorescence staining of LC3II in RAW264.7 cells after different treatments, scale bar = 50 μm. **c** Representative ORO staining images of macrophages with different treatment. **d** ABCA1 and ABCG1 expression levels in different groups. **e** Immunofluorescence staining for iNOS and Arg-1 was performed, scale bar = 50 μm. Data were expressed as the mean ± SD from three independent experiments. Four randomly selected visual fields were counted in each group. *p < 0.05, **p < 0.01

Next, RAW264.7 cells were co-preincubated with the autophagy inhibitor 3-MA or the autophagy inducer Rap to impede or activate autophagic process, respectively. As shown in Fig. 5a, the increased expression of Beclin1 and ratio of LC3II/I and decreased p62 level induced by RVS were significantly blocked by 3-MA, whereas Rap administration elevated Beclin1 expression and LC3II/I ratio and lowered p62 protein abundance. A similar result was observed in immunofluorescence staining of LC3II of the macrophages with different interventions (Fig. 5b).

The ORO staining showed that the burden of lipid accumulation in RAW264.7 cells were alleviated in the RVS-treated group, and this effect were abrogated by 3-MA. On the contrary, Rap treatment effectively diminished ox-LDL-induced lipid deposition in the macrophages (Fig. 5c). Meanwhile, 3-MA blunted the roles of RVS in favoring ABCA1 and ABCG1 expression, and Rap displayed the same effect as RVS on ABCA1 and ABCG1 expression increase (Fig. 5d). Moreover, RVS-induced macrophage polarization to M2 phenotype was also impeded by 3-MA, and Rap displayed the same effect as RVS on regulation of macrophage polarization, as evidenced by immunofluorescence staining of Arg-1 and iNOS (Fig. 5e). There above findings suggested that RVS treatment facilitated lipid efflux and polarization mediation of macrophages through regulating autophagy-related cascades.

### RVS exerted accelerative roles in the autophagy activities of RAW264.7 cells under the stimulation of Chloroquine

**Fig. 6:**
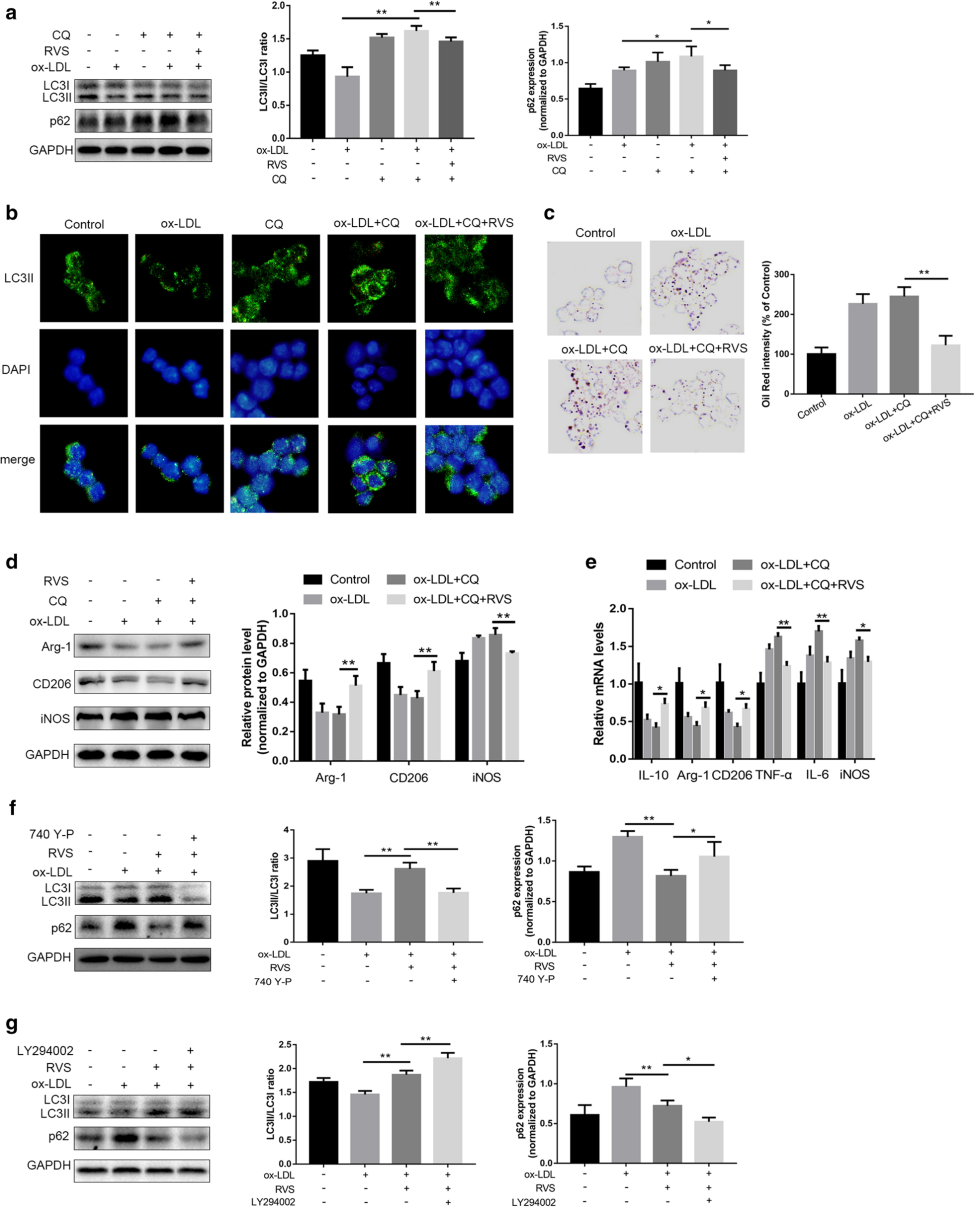
The roles of RVS in the autophagy activities of RAW264.7 insulted by CQ and PI3K/Akt/mTOR signaling pathway regulating autophagy. **a** Representative images of western blot showing LC3 and p62 expression in RAW264.7 cells in different groups. **b** Immunofluorescence staining for LC3II in cells, scale bar = 50 μm. **c** Representative images of ORO-stained lipid droplets. **d** Immunblots and densitometry values of western blot analysis of Arg-1 and iNOS. **e** qRT-PCR mRNA evaluation of genes involved in polarization of macrophages. Effects of **f** PI3K agonist 740 Y-P and **g** PI3K inhibitor LY294002 on LC3II/I ratio and p62 level in ox-LDL-stimulated macrophages. Data were expressed as the mean ± SD from three independent experiments. Four randomly selected visual fields were counted in each group. *p < 0.05, **p < 0.01

CQ exhibited a negative role in fusing autophagosomes and lysosomes and then disrupted the development of autophagic processes [35, 38]. Results of western blot showed that the cells treated with CQ exhibited increased LC3II/I ratio and p62 expression, indicating that CQ blocked autophagy flux (Fig. 6a, b). Furthermore, we observed that CQ accelerated ox-LDL-induced lipid deposition in RAW264.7 cells, whereas which was suppressed by RVS administration (Fig. 6c). In addition, as described in Fig. 6d, e, RVS treatment was capable of antagonizing CQ-triggered inhibition of macrophage polarization to M2-like phenotype under the environment with ox-LDL stimulation, as seen by expression increase of Arg-1, CD206 and IL-10 and level reduction of iNOS, TNF-α and IL-6 in the RAW264.7 cells. These findings hinted that RVS could, to some extent, regulate autophagy flux to affect lipid sedimentation and polarization conversion of macrophages.

### RVS possessed regulatory roles in autophagy activities via mediating the PI3K/Akt/mTOR signaling pathway in RAW264.7 cells

As the above results indicated RVS abated the signal flow of PI3K/Akt/mTOR cascade, we investigated whether RVS improved the autophagy process through affecting the activation of PI3K/Akt/mTOR pathway. As shown in Fig. 6f, we found that the effects of RVS-induced increase of LC3II/I ratio and decrease of p62 level in the macrophages were reversed by intervention of PI3K agonist 740 Y-P. Moreover, Fig. 6g displayed that the PI3K inhibitor LY294002 facilitated elevation of LC3II/I and reduction of p62 content triggered by RVS treatment, which demonstrated that RVS promoted the development of autophagy via abrogating transduction of PI3K/Akt/mTOR pathway.

## Discussion

In this present study, we investigated the potential mechanisms by which RVS affected the processes of lipid deposition and macrophage polarization and then mitigated the progression of atherosclerosis, using in vivo and in vitro models. We discovered that RVS significantly lowered the extent of atherosclerotic plaques and the atheroprotective actions of RVS might be ascribed to regulation of lipid accumulation and polarization transformation of macrophages. Then, we further observed that the upstream mechanisms responsible for anti-atherosclerotic effects of RVS on macrophages were associated with the enhancement of autophagy activities via promoting autophagy flux and suppressing PI3K/Akt/mTOR pathway.

Lipid accumulation and inflammation response in the vascular wall were involved in the occurrence and progression of atherosclerosis [39, 40]. During this pathological process, blood monocytes were considered as an important determiner based on the fact that they migrated into the plaque areas and differentiated into macrophages which could turn to foam cells by swallowing a large amount of lipids and polarize to M1-like phenotype when exposed to the atherosclerotic micro-environment [41]. It was clarified that the imbalance between cholesterol uptake and cholesterol efflux resulted in intracellular cholesterol overloading and foam cell formation [42]. Lipid efflux from macrophages was mainly modulated by membrane transports including ABCA1 and ABCG1, which were responsible for alleviating intracellular lipid accumulation and foam cell formation [43]. We observed that RVS treatment effectively prohibited lipid deposition in plaque lesions, accompanied by expression increase of ABCA1 and ABCG1 in the vascular wall. Similarly, RVS administration significantly reduced the content of lipids and raised the level of ABCA1 and ABCG1 in RAW264.7 cells stimulated by ox-LDL, which suggested that RVS was likely to attenuate lipid sedimentation in macrophages via facilitating the process of lipid efflux. It had been established that stimuli including IFN-γ, LPS and ox-LDL induced macrophages to develop the M1 phenotype which produced pro-inflammatory substances aggravating the progression of atherosclerotic lesions, whereas other factors like IL4 and IL-13 triggered the formation of M2 phenotype that released high levels of anti-atherosclerotic cytokines [44–46]. In this study, we observed that RVS treatment elevated Arg-1 and CD206 expression and reduced iNOS expression in the aortic wall of ApoE^−/−^ mice, which was also seen in ox-LDL-insulted RAW264.7 cells with RVS administration. These data suggested the acceerative roles of RVS in regulating macrophages to display the M2 phenotype which generated anti-inflammatory molecules and then alleviated the development of atherosclerosis.

Autophagy was a highly conserved process involved in clearance of intracellular excessive or defective protein, which exerted regulatory effects on the development of atherosclerosis [47, 48]. Among the bioactive molecules responsible for autophagic activities, Beclin1 mediated the initial step of autophagosome assembly and recruited other autophagy-related genes (Atgs) [49]. The conversion of the cytoplasmic LC3I to the LC3II was an iconic event of autophagy activation, and the LC3II/LC3I ratio was widely used as an indicator to monitor autophagy [49]. Moreover, p62 was an appropriate contributor to recognize and transport discarded cargos to autophagosomes and subsequently degraded along with cargos, which manifested that p62 protein abundance was increased when autophagy flux was blocked [49]. In the present study, our data showed that the LC3II /I ratio and Beclin1 level were significantly increased and p62 level was decreased, and LC3II staining was obviously enhanced in the vascular wall of ApoE^−/−^ mice of RVS group. Similarly, RVS treatment increased the ratio of LC3II/I and Beclin1 level and reduced the content of p62 in ox-LDL-stimulated macrophages, which indicated that RVS effectively enhanced the initiation and development of autophagic process.

There was evidence demonstrating that autophagy activities were capable of weakening the extent of atheroma plaques via promoting cholesterol efflux of macrophages and then prohibiting foam cell formation [15, 50, 51]. Besides, autophagy was reported to modulate the polarization conversion of macrophage phenotype [52]. The disorder of autophagy process had been indicated to be associated with the impairment of lipid clearance and pro-inflammatory phenotype formation of macrophages [52]. Based on this, we hypothesized that RVS mediated the activation of autophagy to encumber lipid accumulation and facilitate M2 phenotypic polarization of macrophages. 3-MA and Rap were used as antagonist and agonist separately and our findings revealed that the beneficial effects of RVS on favoring autophagy implementation was abrogated by 3-MA in RAW264.7 cells insulted by ox-LDL, followed by invalidation of lipid accumulation amelioration and M2-like polarization formation induced by RVS, whereas Rap administration contributed to the positive roles of RVS in autophagy enhancement and subsequent improvement of lipid d and augmentation of generation of M2 phenotype. These observations suggested that RVS treatment exerts atheroprotective effects involving in reduction of foam cell formation and inflammatory phenotype switch via strengthening upstream autophagy processes.

**Fig. 7:**
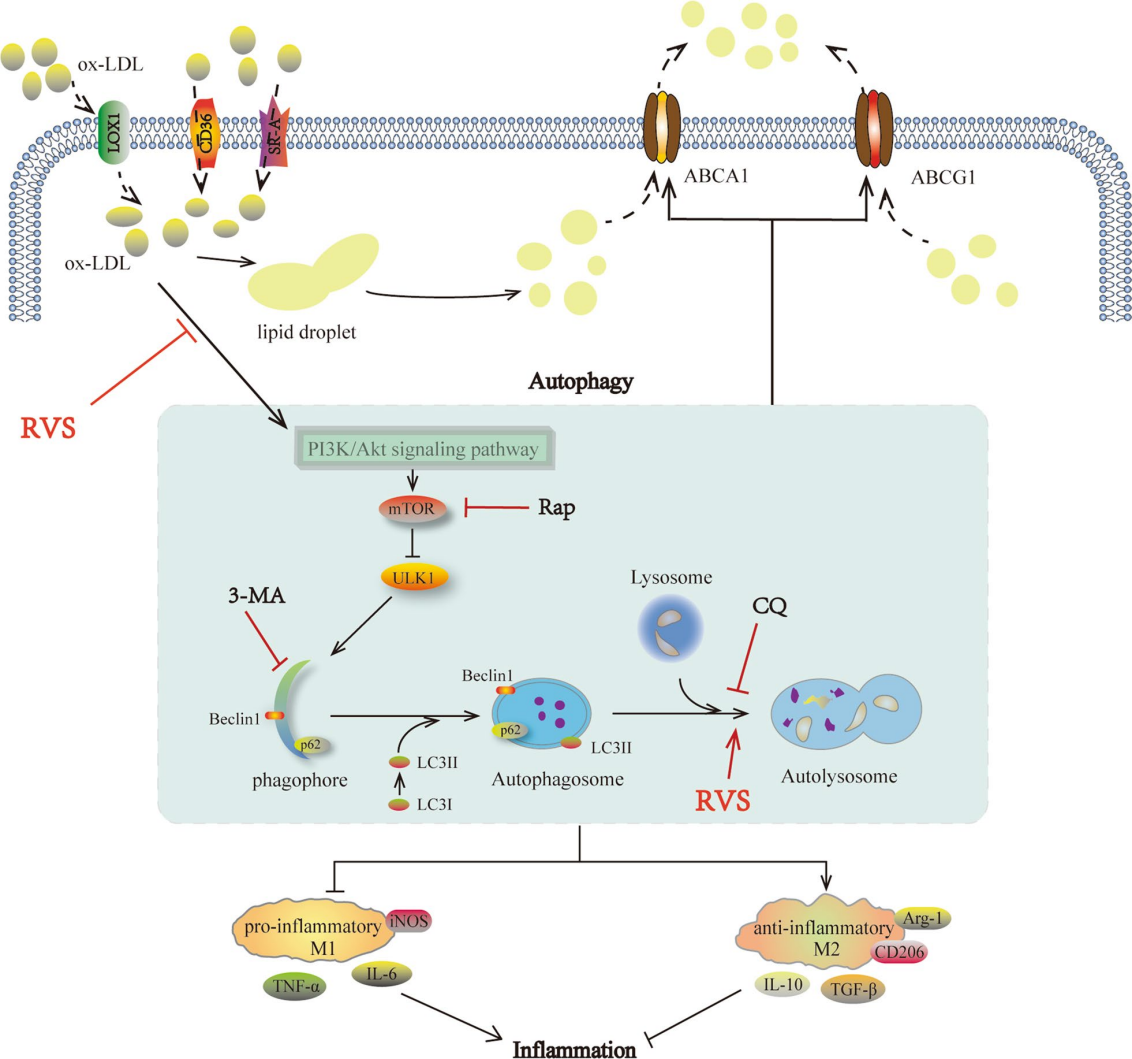
Schematic depiction of anti-atherosclerosis effects of RVS with the key role of autophagy involved in improving lipid accumulation and polarization conversion of macrophages

Then we further investigated relevant mechanisms underlying RVS affected the autophagic activities. CQ, a kind of blocker targeting autophagy flux, was found to increase levels of LC3II/I ratio and p62 content [53], accompanied by deterioration of weakened autophagy in macrophages with ox-LDL intervention, while this effect was significantly alleviated by RVS treatment. PI3K/Akt/mTOR was a classic pathway regulating autophagy initiation and there was evidence revealing that the agents capable of enhancing autophagy via suppressing PI3K/Akt/mTOR pathway reduced the endothelial cell apoptosis induced by oxLDL [54]. Moreover, the inactivation of PI3K/Akt/mTOR pathway was clarified to mediate macrophage autophagy and stabilize the rupture-prone atherosclerotic plaques [21]. Our findings showed that RVS reduced the phosphorylated levels of PI3K, Akt and mTOR and elevated the activity of downstream target ULK1 in vivo and in vitro. Then, we discovered that PI3K agonist 740 Y-P abrogated the beneficial effects of RVS on impelling autophagy-related processes, while PI3K inhibitor LY294002 reinforced RVS-triggered autophagy-promoting effects in RAW264.7 cells stimulated by ox-LDL. These data suggested that RVS increased occurrence and development of autophagy via inhibiting signal transduction of PI3K/Akt/mTOR pathway and improving autophagic flux in macrophages under the lipid-laden condition (Fig. 7).

## Conclusions

In conclusion, the present study indicated that RVS intervention potently inhibited the atherosclerotic plaque development in ApoE−/− mice induced by high-fat diet. Our results provided the evidence that RVS was able to enhance autophagy activities via prohibiting activation of PI3K/Akt/mTOR pathway and increasing autophagic flux, thus leading to the anti-atherosclerotic effects involving suppression of lipid droplets accumulation and facilitation of anti-inflammatory M2 phenotype polarization, which thereby provided novel aspects into the molecular mechanisms of RVS against atherosclerosis development.

## Data Availability

The data that support the findings of this study are available from the corresponding author upon reasonable request.

## Abbreviations

RVS: Rosuvastatin
ApoE^−^^/^^−^: Apolipoprotein E-deficient
ox-LDL: Oxidized low-density lipoprotein
mTOR: Mammalian target of rapamycin
HMG-CoA: 3-Hydroxyl-3-methylglutaryl coenzyme A
HE: Hematoxylin and eosin
TG: Triglyceride
TC: Total cholesterol
LDL-C: Low density lipoprotein cholesterol
HDL-C: High density lipoprotein cholesterol
ATCC: American Type Culture Collection
DMEM: Dulbecco’s modified Eagle medium
3-MA: 3-Methyladenine
Rap: Rapamycin
CQ: Chloroquine
ORO: Oil Red O
SD: Standard deviation
Atgs: Autophagy-related genes
